# Quantitative Proteomic Analysis Reveals Antiviral and Anti-inflammatory Effects of Puerarin in Piglets Infected With Porcine Epidemic Diarrhea Virus

**DOI:** 10.3389/fimmu.2020.00169

**Published:** 2020-02-26

**Authors:** Mengjun Wu, Qian Zhang, Dan Yi, Tao Wu, Hongbo Chen, Shuangshuang Guo, Siyuan Li, Changzheng Ji, Lei Wang, Di Zhao, Yongqing Hou, Guoyao Wu

**Affiliations:** ^1^Hubei Key Laboratory of Animal Nutrition and Feed Science, Wuhan Polytechnic University, Wuhan, China; ^2^Department of Animal Science, Texas A&M University, College Station, TX, United States

**Keywords:** puerarin, porcine epidemic diarrhea virus, piglets, antiviral, anti-inflammatory, proteomics

## Abstract

Porcine epidemic diarrhea virus (PEDV) has caused enormous economic losses to the swine industry worldwide in recent years. Puerarin (PR), a major isoflavonoid isolated from the Chinese herb Gegen, possesses many pharmacological activities, including anti-inflammatory, and anti-viral activities. This study was conducted with both PEDV-infected African green monkey kidney cells (Vero) and neonatal pigs to determine the effect of PR on PEDV infection and to elucidate the underlying mechanisms by using proteomic analyses. Twenty-four piglets fed a milk replacer were randomly allocated into one of three groups (Control, PEDV, and PEDV + PR). After a 5-day period of adaption, piglets (*n* = 8/group) in the PEDV + PR were orally administered with PR (0.5 mg/kg body weight) between days 5 and 9, whereas piglets in the other two groups received the same volume of liquid milk replacer. On day 9, piglets were orally administered with either sterile saline or PEDV (Yunnan province strain) at 10^4.5^ TCID_50_ (50% tissue culture infectious dose) per pig. On day 12 of the trial, jugular vein blood and intestinal samples were collected. In addition, Vero cells were assigned randomly into three groups (Control, PEDV, PEDV + PR). Cells in the PEDV and PEDV + PR groups were infected with PEDV at a multiplicity of infection of 0.01, while cells in the control group were treated with the same volume of sterile saline. One hour later, cells in the Control and PEDV groups were cultured in serum-free DMEM, while cells in the PEDV + PR group were supplemented with PR. After 36 h of culture, cells were harvested. PR attenuated the reductions in cell proliferation *in vitro* and growth performance in PEDV-infected piglets, and inhibited PEDV replication and the expression of several cytokines (including IL-8) both *in vitro* and *in vivo*. Proteomic analyses identified that the abundances of 29 proteins in the ileum were altered by PEDV infection and restored to the control level by PR. Pathway analyses revealed that PR restored the expression of several interferon-stimulated genes and selectively upregulated the expression of guanylate-binding proteins. Western blot analyses showed that PR supplementation inhibited the PEDV-induced NF-κB activation. Collectively, these results indicate that PR could exert antiviral and anti-inflammatory effects in piglets infected with PEDV and have the potential to be an effective antiviral feed additive.

## Introduction

Porcine epidemic diarrhea (PED), a devastating enteric disease characterized by vomiting, anorexia, acute severe watery diarrhea, and dehydration, results in extremely high rates of morbidity and mortality in newborn piglets (1). Since December 2010, massive outbreaks of PED have occurred in China, with 80–100% morbidity and 50–90% mortality in suckling piglets (2, 3). The recent research provides evidence for airborne transmission of PED virus (PEDV) (4), which has greater transmission potential than other seasonal diarrhea viruses (5, 6). The small intestine, especially the jejunum and ileum, is the target tissue of PEDV infection (7). The M Protein and the N protein, being the most abundant envelope component and a structural basis for the helical nucleocapsid of PEDV, respectively, are commonly used in the clinical diagnosis of PED (8–10). To date, no feed additive is available to effectively inhibit the replication of PEDV.

Owing to the concerns about the toxicity of synthetic antiviral drugs, natural products are considered to be an important source of new drug development against viral infections (11). Puerarin (PR) is an isoflavonoid isolated from Gegen, which is a traditional Chinese herb medicine (12). PR has anti-oxidant and anti-inflammatory effects (13–15) and has been proven to be an effective antimicrobial agent (16). PR significantly prevented human alveolar epithelial A549 cells from *Staphylococcus aureus*-induced injury and may be considered as a potential candidate for the development of anti-virulence drugs in the treatment of *S. aureus*-mediated infections (17). A recent study found that pre-treatment with PR protected porcine intestinal epithelial cells (IPEC-J2 cells) from enterotoxigenic *Escherichia coli* (ETEC) infection through inhibiting bacterial adhesion and inflammatory responses (18). Another study found that PR had a moderate ability to reduce hepatitis B virus production *in vitro* (19). Additionally, *Kudzu* root-extracted PR inhibited HIV-1 replication by blocking the initial attachment of the viral particle to the cell surface in primary human CD4^+^ T lymphocytes and macrophages (20). Lin et al. found that the water extract of *Pueraria lobata* Ohwi has anti-viral activity against human respiratory syncytial virus in human respiratory tract cell lines (21). Therefore, these results suggest that PR could be a promising supplement for antiviral therapy. However, little is known about its effectiveness against PEDV infection.

Proteomic analysis is widely used in biomedical science for discovering novel molecular interactions and pathways (22). The label-free quantitative proteomic (LFQP) analysis is a very powerful tool to profile global protein expression (23). Bioinformatics analysis has been applied to explore the mechanisms of interaction among the host, pathogen, and drug. In recent years, the quantitative proteomic analysis has been used to investigate the pathogenic mechanism of PEDV infection (24, 25). However, most of these studies are performed *in vitro* with a label-based quantitative proteomic (LQP) approach. Previous studies have indicated that the label-free approach by far outperforms the LQP method for the proteome coverage, as up to threefold more proteins are reproducibly identified in replicate measurements (26). It is of great importance to study the interaction between PEDV and the host *in vivo*, which could provide more biologically relevant insights into the pathogenesis of PEDV.

In this study, the antiviral effect of PR was evaluated in PEDV-infected Vero cells and neonatal pigs. Furthermore, an LFQP analysis was used to identify differentially regulated proteins (DRPs) in the small intestine and the mechanisms underlying the effects of PR on PEDV infection. Our findings are expected to provide a basis for the use of PR to treat PEDV infection.

## Materials and Methods

### PEDV, Vero Cells, and PR

PEDV (Yunnan province strain, GenBank accession No. KT021228) and African green monkey kidney cells (Vero) were provided by the State Key Laboratory of Agricultural Microbiology, College of Veterinary Medicine, Huazhong Agricultural University, Wuhan, China. PR (purity ≥ 98%) was purchased from Macklin Inc. (Macklin, Shanghai, China).

### Viral Infection in Vero Cells

All experiments using live virus *in vitro* were conducted under biosafety level 2 (BSL2) conditions and strictly followed safety procedures. Vero cells were randomly assigned into three groups (Control, PEDV, PEDV + PR) and cultured in Dulbecco's modified Eagle's medium (DMEM, Tanee Chemicals, Beijing, China) supplemented with 10% fetal bovine serum (FBS; Gibco; Thermo Fisher Scientific, Inc., Waltham, MA, USA), and 1% penicillin–streptomycin–amphotericin B (PSF; Solarbio, Beijing, China) in 75-cm^2^ flasks under the condition of 5% CO_2_ at 37°C. Upon reaching 80–90% confluency, the cells were washed twice gently with serum-free DMEM prior to infection. Then the cells were cultured with PEDV at a multiplicity of infection (MOI) of 0.01 in serum-free DMEM containing 5 μg/ml of trypsin (Genom, Hangzhou, China). Cells without PEDV infection were used as the Control. One hour later, the Control and PEDV groups were replenished with 100 μl of serum-free DMEM, whereas the PEDV + PR group was supplemented with PR. After 36 h of incubation, the cells were washed twice with phosphate-buffered saline (PBS; Gibco, USA) and harvested for further analysis.

### Animals and Treatments

The animal use protocol for this research was approved by the Animal Care and Use Committee of Wuhan Polytechnic University (Index number: 011043145-029-2013-000009). Twenty-four 7-day-old crossbred (Duroc × Landrace × Large White) healthy piglets (half male and half female), initially weighing 3.17 ± 0.25 kg, were purchased from a PEDV-negative farm. They were randomly allocated into one of three treatment groups (Control, PEDV, PEDV + PR; eight replicates per group). The experimental basal diet (a liquid milk replacer), which was formulated to meet the requirements of all nutrients for suckling piglets, was purchased from Wuhan Anyou Feed Co., Ltd. (Wuhan, China). Piglets were housed in clean pens with strict control of cross-infection. The entire trial period was 12 days. During day 5 to 9 of the trial, the piglets in the PEDV + PR group were orally administered with PR (0.5 mg/kg body weight; dissolved in the liquid milk replacer), and the piglets in the other two groups received the same volume of the liquid milk replacer. On day 9 of the trial, PEDV at a dose of 10^4.5^ TCID_50_ (50% tissue culture infectious dose) per pig was orally inoculated to pigs in the PEDV and the PEDV + PR groups, while the Control group was mock inoculated in parallel with the same volume of sterile saline. Pigs were observed daily to record the health status and diarrhea incidence. On day 12, all piglets were weighed and sacrificed to obtain the jejunum, ileum, colonic chyme, and mesenteric lymph nodes. All samples were rapidly frozen in liquid nitrogen and then stored at−80°C until analysis. The protocols of PEDV infection and sample collection were described previously (27). The intestinal pathomorphology was observed as described previously (28).

### Quantitative RT-PCR (qRT-PCR) and Droplet Digital PCR (dd PCR)

The total RNA was extracted by TRIzol reagent (Takara, Dalian, China) to ensure its purity (a 28 S/18 S rRNA ratio of >1.8 and an OD_260_/OD_280_ ratio of approximately 2.0) according to the manufacturer's instructions. The cDNA was synthesized by RT-PCR using the PrimeScript® RT reagent Kit with gDNA Eraser kit (Takara, Dalian, China). The qPCR was performed using the SYBR® Premix Ex TaqTM (Takara, Dalian, China) on an Applied Biosystems 7,500 Fast Real-Time PCR System (Foster City, CA, USA). The relative expression level of each gene was calculated with the 2^−ΔΔCT^ method (29). Monkey hypoxanthine phosphoribosyltransferase 1 (HPRT1) was used as the reference gene in Vero cells (30). Porcine ribosomal protein L4 (RPL4) was used as the reference gene in the jejunum, ileum, and mesenteric lymph nodes (28). The genomic DNA of the colon chyme was extracted by using the QIAamp® Fast DNA Stool Mini Kit (Qiagen, Hilden, Germany). Then the viral gene expression was detected by using the droplet digital PCR (dd PCR) method as described previously (31). Primers used in the present study are listed in Table 1 (32–35).

**Table 1:** The primer sequences used in the present study.

**Species**	**Gene name**	**Sequences**	**References**
Monkey	HPRT1	F:5′-TGACACTGGCAAAACAATGCA-3′	(30)
		R:5′-GGTCCTTTTCACCAGCAAGCT-3′	
	IL-1β	F:5′-GCGGCAACGAGGATGACTT-3′	(32)
		R:5′-TGGCTACAACAACTGACACGG-3′	
	IL-8	F:5′-GGAACCATCTCGCTCTGTGTAA-3′	(32)
		R:5′-GGTCCACTCTCAATCACTCTCAG-3′	
	TNF-α	F:5′-CACCACGCTCTTCTGTCT-3′	(32)
		R:5′-AGATGATCTGACTGCCTGAG-3′	
	MCP-1	F:5′-CTTCTGTGCCTGCTGCTCATA-3′	(32)
		R:5′-ACTTGCTGCTGGTGATTCTTCT-3′	
Pig	RPL4	F:5′-GGAAACCGTCGCGAGA-3′	(28)
		R:5′-GCCCCAGAGACAGTT-3′	
	GBP-2	F:5′-ACCAGGAGGTTTTCGTCTCTCTATT-3′	Present study
		R:5′-TCCTCTGCCTGTATCCCCTTT-3′	
	IFIT3	F:5′-GCATTTTCCAGCCAGCATC-3′	(33)
		R:5′-TCTGTTCCTTTCCTTTCCTTCCT-3′	
	OAS1	F:5′-TGGTGGTGGAGACACACACA-3′	(33)
		R:5′-CCAACCAgAgACCCATCCA-3′	
	IFN-α	F:5′-ACTCCATCCTGGCTGTGAGGAAAT-3′	(33)
		R:5′-ACTCCATCCTGGCTGTGAGGAAAT-3′	
	IFN-β	F:5′-ATGTCAGAAGCTCCTGGGACAGTT-3′	(34)
		R:5′-AGGTCATCCATCTGCCCATCAAGT-3′	
PEDV	PEDV-M	F:5′-TCCCGTTGATGAGGTGAT-3′	Present study
		R:5′-AGGATGCTGAAAGCGAAAA-3′	
	PEDV-N	F:5′-CGCAAAGACTGAACCCACTAACTT-3′	(35)
		R:5′-TTGCCTCTGTTGTTACTCGGGGAT-3′	

*Abbreviations: HPRT1, hypoxanthine phosphoribosyltransferase 1; RPL4, ribosomal protein L4; IL-1β, interleukin-1β; IL-8, interleukin-8; TNF-α, tumor necrosis factor α; MCP-1, monocyte chemotactic protein 1; PEDV-M, membrane protein (porcine epidemic diarrhea virus); PEDV-N, nucleocapsid protein (porcine epidemic diarrhea virus); GBP-2, guanylate-binding proteins; IFIT3, interferon-induced protein 3; OAS1, 2′-5′oligoadenylatesynthetase 1; IFN-α, interferon-α; IFN-β, interferon-β*.

### Cytokine Assays by Enzyme-Linked Immunosorbent Assay (ELISA)

The concentrations of cytokines (IL-1β, IL-6, IL-8) in the ileum of piglets were measured by using ELISA kits (RD Systems Quantikine, USA), which are specific for porcine cytokines. All the assay procedures were performed according to the manufacturer's instructions.

### Protein Extraction and Digestion

Four biological replicates per group, i.e., a total of 12 ileal samples, were used to analyze the intestinal proteome. First, 150 mg of ileal tissue was homogenized with 1 ml of Tissue Protein Extraction Reagent (Thermo Scientific, USA) containing Protease Inhibitor (Roche, USA) and then vortexed for 10 min at 4°C. The soluble protein was collected after the removal of tissue debris by centrifugation (12,000 × g, 20 min, 4°C). The protein concentration was quantified by the bicinchoninic acid (BCA) assay (25). Approximately 200 μg of proteins from each sample was subjected to filter-aided sample preparation (FASP) trypsin digestion to generate peptides. Briefly, protein samples were diluted to 100 μl with 50 mM ammonium bicarbonate, and disulfide bonds in protein were reduced by treating the sample with 2 μl of 500 mM DL-dithiothreitol (DTT) in 100 μl of 50 mM ammonium bicarbonate for 150 min at 37°C (the final concentration of DDT was 10 mM), and then alkylated with 14 μl of 500 mM iodoacetamide (IAA) at room temperature (25°C) for 40 min in the dark (the final concentration of IAA was 60 mM). The detergent, DTT, and other low-molecular-weight components were removed by repeated dilution in 100 mM Tris-HCl UA buffer (containing 8 M urea, pH 8.5), followed by filtration through 10-kDa ultrafiltration units (Pall Corporation, USA). The filters were washed three times with 100 μl of UA buffer, followed by twice washes with 100 μl of 50 mM ammonium bicarbonate. Proteins were then digested using mass spectrometry-grade trypsin (Thermo Fisher Scientific, USA) in 50 mM ammonium bicarbonate by incubation overnight at 37°C and 800 rpm. The enzyme:substrate ratio was 1:50. The effluent was collected after centrifugation at 10,000 × g for 15 min and filtered twice with 50 μl of 50 mM ammonium bicarbonate. The effluent was combined and then acidified with 10% trifluoroacetate (TFA) to stop the reaction (the final concentration of TFA was 0.4%). The resulting peptides were desalted using a Pierce C18 Tips (Thermo Fisher Scientific, USA) and vacuum-dried. Prior to LC-MS/MS analysis, peptide samples were re-suspended in 40 μl of 0.1% v/v formic acid (FA) and 5% v/v acetonitrile (ACN) solution.

### Nano-LC-MS/MS Analysis

The nano-LC-MS/MS analysis was performed in 125 min on a Q Exactive mass spectrometer (Thermo Fisher Scientific, USA), which was coupled to an Easy-Nano Ultimate 3,000 UPLC system (Dionex, Thermo Fisher Scientific, USA). The peptide mixture (1 μg) was loaded into a trap column (100 μm × 20 mm; Acclaim PepMap 100; Thermo Fisher Scientific, USA) at 5 μl/min for 3 min. Peptide separation was carried out in a C18 capillary column (75 μm × 150 mm; Acclaim PepMap RSLC, Thermo Fisher Scientific, USA) at 300 nl/min. The mobile phase consisted of two solvents: 0.1% formic acid (FA) in water (solvent A) and 0.08% FA in 80% acetonitrile (ACN, solvent B). The following gradients were used: 0–3% B in 8 min, 3–8% B in 2 min, 8–28% B in 76 min, 28–45% B in 17 min, 45–99% B in 2 min, 99% B in 5 min, 99–3% B in 1 min, 3% B in 14 min. Then the separated peptides were analyzed in the Q Exactive Orbitrap mass spectrometer operated in the positive ion mode (nanospray voltage was 2.0 kV and source temperature was 250°C). The MS data were acquired using the data-dependent acquisition (DDA) mode. The mass spectrometer was operated in the Top-20 data-dependent mode with automatic switching between MS/MS and the full-scan MS mode (350–2,000 m/z) that was operated at a resolution of 70,000 with automatic gain control (AGC) target of 3 × 10^6^ ions and a maximum ion transfer (IT) of 20 ms. The precursor ions were fragmented by high-energy collisional dissociation (HCD) and subjected to MS/MS scans with the following parameters: resolution, 17,500; fixed first mass, 120 m/z; isolation width, 1.6 m/z; AGC, 1 × 10^5^ ions; maximum IT, 50 ms; intensity threshold, 1.6 × 10^5^; normalized collision energy, 27%; dynamic exclusion duration, 40.0 s. These data were acquired by the Xcalibur software (version 4.0.27.10, Thermo Fisher Scientific, USA).

### MS Data Analysis

The peak lists extracted from raw MS data were further processed with the MaxQuant software (version 1.6.1.0, Max Planck Institute of Biochemistry in Martinsried, Germany) by using the XIC (extracted ion chromatogram)-based label-free quantitation algorithm and the UniProtKB *Sus scrofa* database (22,191 total entries, downloaded 11/10/18). The M and N proteins were analyzed similarly on the basis of PEDV CV777 strain database (seven total entries, downloaded 11/10/18). The following parameters were applied for the search: enzyme, trypsin; missed cleavages, 2; minimum peptide length, 7; de-isotopic, TRUE; fixed modification, carbamidomethyl (C), variable modification, oxidation (M) and acetylation (protein N-terminus); decoy database pattern, reverse; label-free quantification (LFQ), TRUE; LFQ minimum ratio count, 2; iBAQ, TRUE; match between runs, 2 min; peptide false discovery rate (FDR) ≤ 0.01; protein FDR ≤ 0.01. MaxQuant output files were analyzed with standard settings in the Perseus software (v.1.6.2.3, http://www.perseus-framework.org) (36). LFQ intensity values were used as the quantitative measurement of protein abundance for subsequent analysis. The data matrix was first filtered for the removal of contaminants and reverse decoy data. Each of the confident protein identification involves at least two unique peptides. LFQ intensity values were log2 transformed (37), and each sample was assigned to its corresponding group (Control, PEDV, or PEDV + PR). Proteins were filtered based on valid values (Min. number of values: four, Mode: in total). A data-imputation step was conducted to replace missing values with those that simulate signals of low abundant proteins (38). The latter were chosen randomly from a distribution specified by a downshift of 1.8 times the standard deviation (SD) of all measured values and a width of 0.3 times the SD. Two sample *t*-tests were performed for all relevant comparisons using a cutoff of *P* < 0.05 on the post imputation datasets to identify statistically significant differentially regulated proteins (DRPs).

### Bioinformatics Analysis

Heat-map was used to show the trend of protein expression between different samples with the aid of Metaboanalyst (https://www.metaboanalyst.ca/faces/home.xhtml). All those proteins that exhibited a fold-change of at least 1.5 (*P* < 0.05) were considered differentially regulated. The protein names were obtained from UniProt gene ID using the UniProt “Retrieve/ID mapping” function (http://www.uniprot.org/uploadlists/). For functional analysis, Gene Ontology (GO) annotation was retrieved from the GO database (http://www.geneontology.org/) for each protein in search of the database. Due to the poor annotations of the porcine genome database, the differentially expressed proteins were input into the human database for the Reactome pathway analysis (http://www.reactome.org). In this study, cell signaling pathways were selected with the threshold of significance being defined as *P* < 0.05, FDR < 0.05, and minimum counts > 3. The relationships among the DRPs of the three groups were depicted in Venn diagrams (http://bioinformatics.psb.ugent.be/webtools/Venn/). Furthermore, protein–protein interaction networks were constructed using the online software String (https://string-db.org/) in combination with Cytoscape (v.3.7.1, https://cytoscape.org/).

### Validation of the Proteomic Analysis Results by Western Blot

The Western blot assay was carried out as previously described (39) to validate the results of proteomic study. In brief, proteins were extracted, denatured, and quantified using the BCA assay kit (Thermo Fisher Scientific, USA). Equivalent quantities of proteins from the independent biological replicates were separated by 10% SDS-PAGE, and the separated proteins were then electrophoretically transferred onto polyvinylidene difluoride (PVDF) membranes, which were subsequently blocked with 5% w/v skim milk in Tris-buffered saline containing Tween 20 (TBST) for 1.5 h at 25°C. Thereafter, the membranes were incubated at 4°C overnight with one of the following primary antibodies: signal transducers and activators of transcription 1 (STAT1; 1:1,000; Nova Biologicals), IFN-stimulated genes 15 (ISG15; 1:1,000; Abcam), myxovirus resistance 1 (MX1; 1:1,000; Abcam), retinoic acid-inducible gene 1 (DDX58; 1:1,000; Cell Signaling Technology), nuclear factor kappa-B (NF-κB p65; 1:1,000; Cell Signaling Technology), and phosphorylated-nuclear factor kappa-B (pNF-κB p65; 1:1,000; Cell Signaling). After washing three times with TBST, the membranes were incubated with the anti-rabbit (mouse) immunoglobulin G horseradish peroxidase-conjugated secondary antibody (1:5,000 dilution; Beijing ZhongShan Golden Bridge Biological Technology Co. Ltd, Beijing, China). The abundance of β-actin (1:4,000; Invitrogen) in each sample was also determined as an internal reference in the present study, whereas the histone H3 protein was determined as an internal reference for nuclear proteins.

### Statistical Analysis

The data of ADG, morphology, qRT-PCR, and Western blot were analyzed by using the one-way ANOVA procedure in the SPSS 17.0 software (SPSS Inc. Chicago, USA) and expressed as mean ± SD. The differences between means among the treatment groups were determined by the Duncan's multiple range test. In addition, data of the diarrhea rate were analyzed by the Chi-square test and expressed as a percentage. A value of *P* < 0.05 was taken to indicate statistical significance.

## Results

### Effects of PR on PEDV Replication and PEDV-Induced Cytokine Expression in Vero Cells

**Figure 1:**
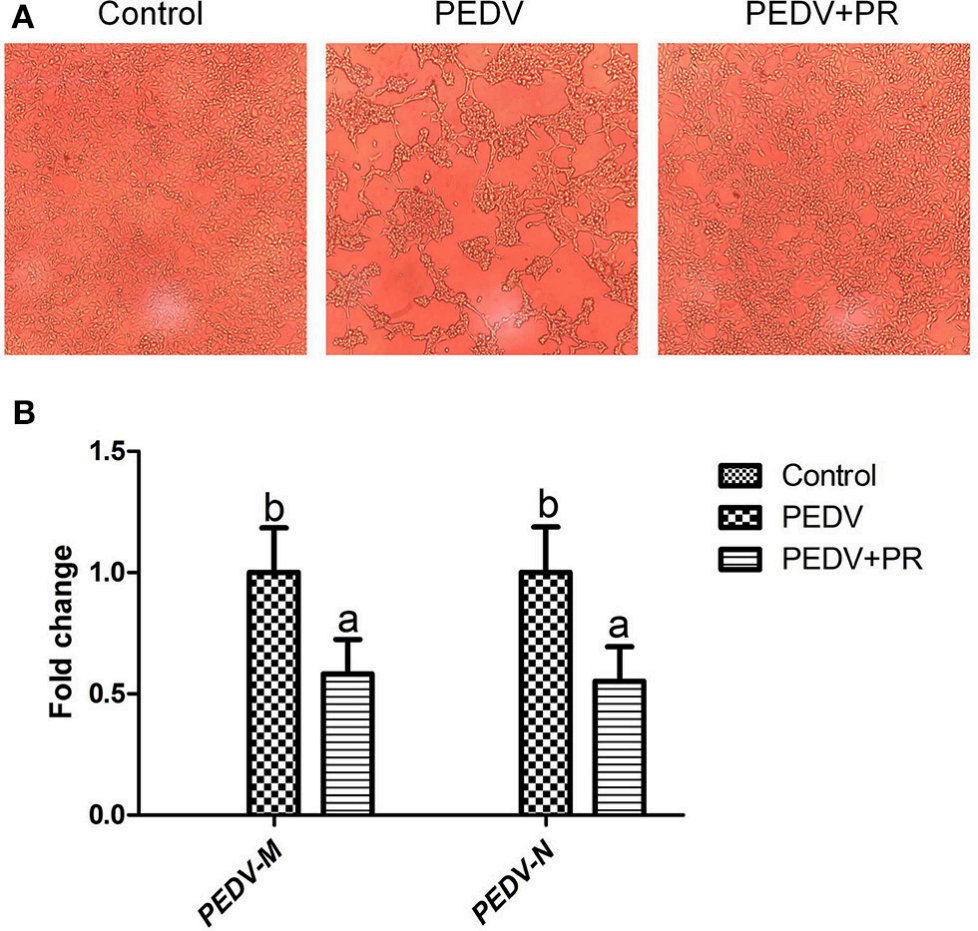
Effects of puerarin on porcine epidemic diarrhea virus (PEDV) replication in Vero cells. **(A)** Microscopic morphology of Vero cells in culture (100×). Typical cytopathic effects (CPE) of PEDV in Vero cells were revealed by an optical microscope at 36 h post inoculation. Cell detachment appeared in the PEDV group compared to the Control group. However, the PEDV + PR group appeared to have fewer dead cells than the PEDV group. **(B)** Effects of puerarin on the expression of *M* and *N* genes of PEDV in Vero cells. Values are mean and pooled SEM, *n* = 8. ^a,b^Values with different letters for the same gene are different (*P* < 0.05).

**Figure 2:**
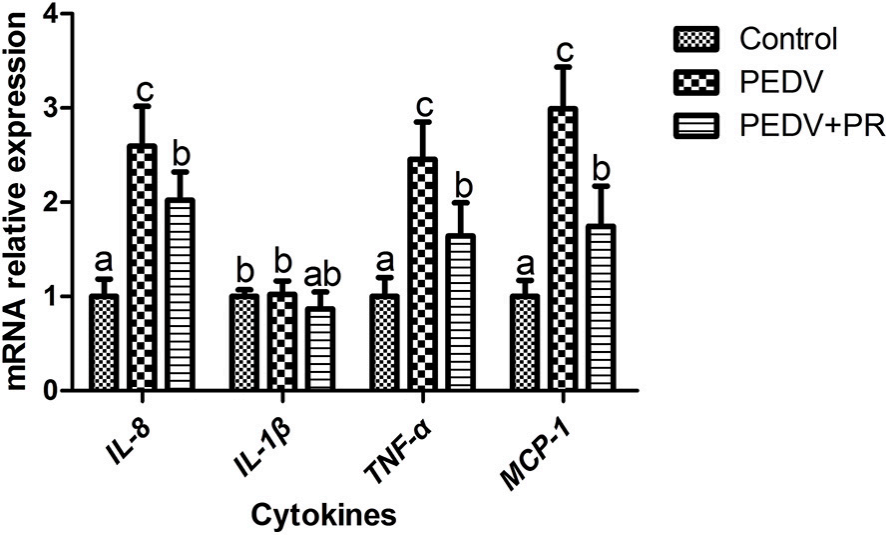
Effects of puerarin on the expression of PEDV-induced cytokines in Vero cells. Values are mean and pooled SD, *n* = 8. ^a,b,c^Values with different letters for the same gene are different (*P* < 0.05).

To assess the role of PR on PEDV infection, an *in vitro* study was performed with Vero cells. Microscopic observation revealed that cells died and shed from the wall after PEDV infection (Figure 1A). However, cells in the PEDV + PR group exhibited a lower rate of deaths than the PEDV group (*P* < 0.05). qPCR results showed that the mRNA levels of PEDV *N* and *M* genes were lower in the PEDV + PR group than those in the PEDV group (Figure 1B). Moreover, the expression of *IL-8, TNF-*α, and *MCP-1* genes were decreased (*P* < 0.05) in the PEDV + PR group compared with the PEDV group (Figure 2).

### Effects of PR on Resistance to PEDV Infection in Piglets

**Figure 3:**
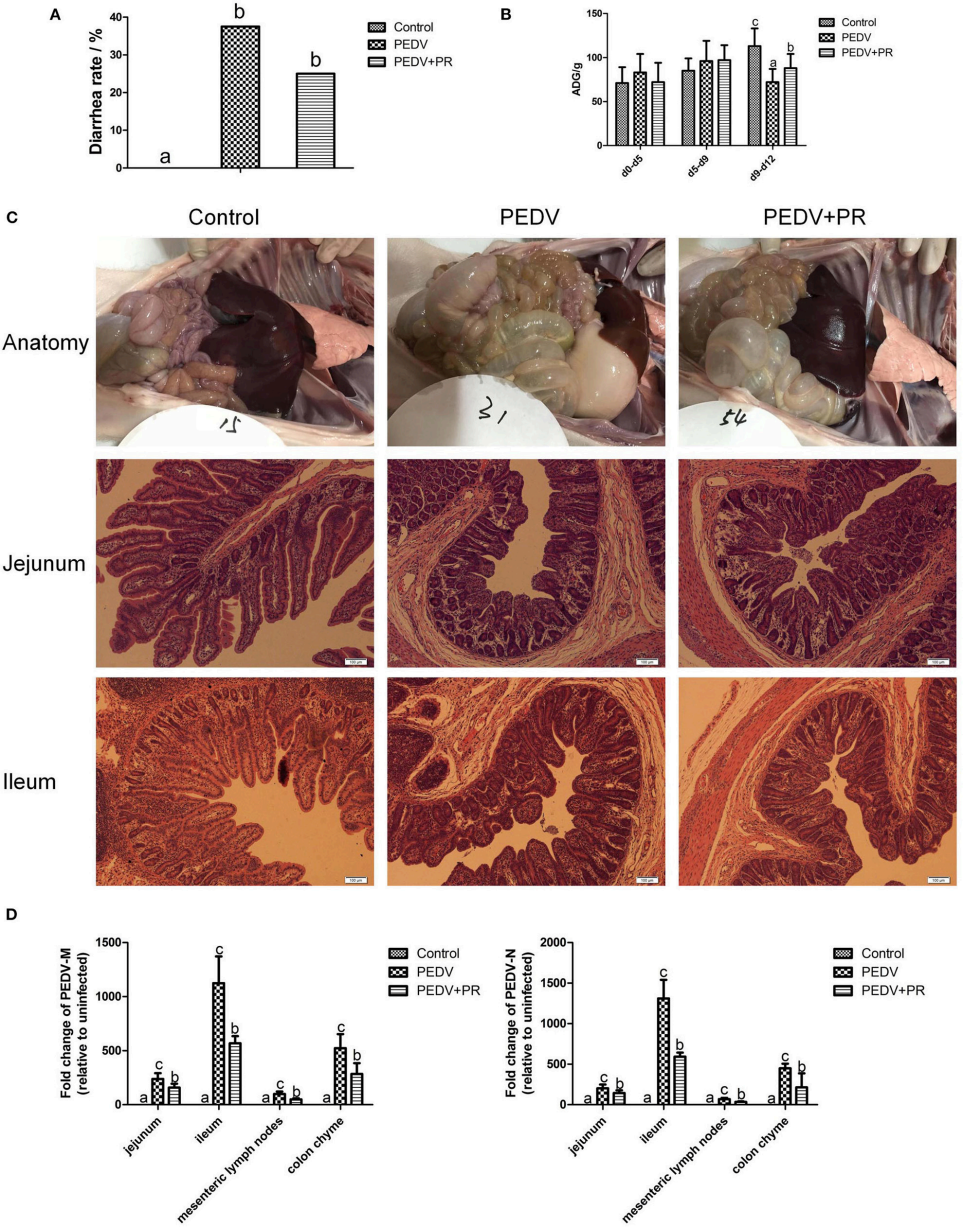
Effects of puerarin on resistance in PEDV-infected piglets. **(A)** Effects of puerarin on the diarrhea incidence of piglets during PEDV infection. **(B)** Effects of puerarin on the average daily gain of piglets during PEDV infection. ADG, average daily weight gain. d0–d5: No treatment. d5–d9: Piglets in the PEDV + PR group were orally administered with puerarin (prepared in liquid milk replacer), and the other two groups of piglets received the same volume of liquid milk replacer. d9–d12: On day 9 of the experiment, 3.3 ml of PEDV were orally inoculated to the PEDV group and the PEDV + PR group, whereas the Control group received the same volume of saline. **(C)** Histopathological structures of piglet intestines in the Control, PEDV, and PEDV + PR groups. PEDV infection caused typical PED symptoms. Piglets in the PEDV group exhibited moderately thin and transparent intestinal walls with an accumulation of large amounts of fluid in the intestinal lumen. Pathological examination of the jejunum and ileum of piglets using the hematoxylin and eosin (H&E) staining revealed multifocal to diffuse villous atrophy. However, piglets in the PEDV + PR group exhibited less intestinal lesions than those in the PEDV group. **(D)** Effects of puerarin on the expression of *PEDV-M* and -*N* genes in different tissues of piglets. Values are mean and pooled SD, *n* = 8. ^a,b,c^Values with different letters for the same tissue are different (*P* < 0.05).

In this study, piglets in both PEDV and PEDV + PR groups exhibited watery diarrhea after PEDV infection. Diarrhea incidence in the PEDV + PR group tended to decrease in comparison with the PEDV group, but the difference did not reach statistical significance (Figure 3A). Moreover, other clinical symptoms, such as vomiting, anorexia, and reduced appetite, were mitigated in the PEDV + PR group in comparison with those in the PEDV group (data not shown). Prior to PEDV challenge (day 0–day 5 and day 5–day 9), average daily weight gain (ADG) did not differ among the three groups of piglets (Figure 3B). However, PEDV infection reduced (*P* < 0.05) the ADG of piglets (day 9–day 12), and oral administration of PR alleviated the reduction of ADG in PEDV-infected piglets (*P* < 0.05).

In the present study, the small intestine was typically distended by the accumulation of yellow fluid, and the small intestinal walls were thin and transparent in the PEDV and PEDV + PR groups. Histologic lesions were present in the jejunum and ileum of PEDV-infected groups, including the irregularity and desquamation of epithelial cells, as well as the defected and irregular striated border (Figure 3C). No pathological changes were observed in the small intestine of the control group (non-infected). Due to the severe villous atrophy in PEDV-infected piglets, villus height (VH), villus surface area, crypt depth (CD), and the ratio of VH/CD were further measured. Results showed that crypt depths were increased, whereas villus height, villus surface area, as well as the ratio (VH/CD) of jejunum and ileum were decreased (*P* < 0.05) in both PEDV and PEDV + PR groups. No statistical difference in the intestinal morphology was found between PEDV and PEDV + PR groups (Table 2).

**Table 2:** The intestinal morphology of piglets.

**Items**	**Control**	**PEDV**	**PEDV + PR**
**Villus height (μm)**			
Jejunum	309.63 ± 72.97^b^	112.45 ± 17.07^a^	114.13 ± 16.16^a^
Ileum	251.27 ± 53.59^b^	144.27 ± 24.78^a^	116.04 ± 13.55^a^
**Crypt depth (μm)**			
Jejunum	133.35 ± 14.08^a^	172.04 ± 26.78^b^	161.41 ± 21.94^b^
Ileum	132.78 ± 14.82^a^	161.16 ± 21.45^b^	159.92 ± 29.25^b^
**Villus height/crypt depth**			
Jejunum	2.37 ± 0.81^b^	0.68 ± 0.10^a^	0.76 ± 0.13^a^
Ileum	1.96 ± 0.55^b^	0.94 ± 0.16^a^	0.81 ± 0.13^a^
**Villus surface area (μm**^**2**^**)**			
Jejunum	7372.09 ± 1901.61^b^	2735.40 ± 570.41^a^	2734.74 ± 454.43^a^
Ileum	6751.92 ± 1895.39^b^	3431.66 ± 695.99^a^	2885.31 ± 173.09^a^

Values are mean and pooled SD, n = 8.

a,b *Values within a row not sharing a common superscript letter are different (P < 0.05)*.

To further determine the role of PR on PEDV infection *in vivo*, the mRNA levels for PEDV *M* and *N* genes were measured. The *M* and *N* genes were highly expressed in both PEDV and PEDV + PR groups, whereas no PEDV genes were detected in the Control group. These results indicated that the PEDV infection model was successfully established in this study. However, compared with the PEDV group, mRNA levels for the PEDV *M* and *N* genes were decreased (*P* < 0.05) in the jejunum, ileum, mesenteric lymph nodes, and the colon of PEDV + PR piglets (Figure 3D).

### Effects of PR on PEDV-Induced Cytokine Expression *in vivo*

**Figure 4:**
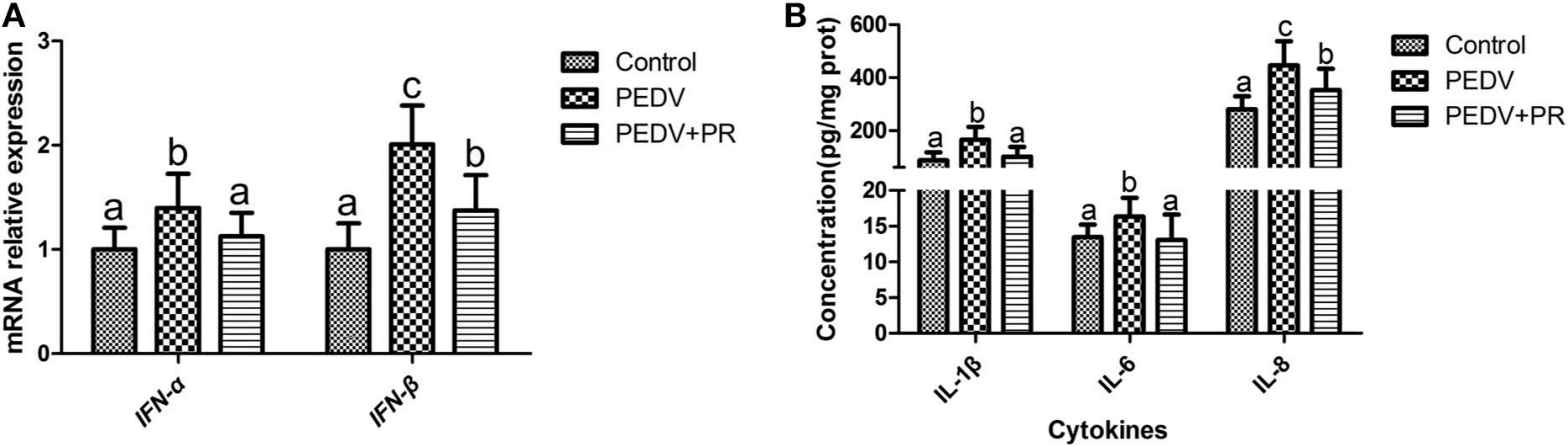
Effects of puerarin on the expression of PEDV-induced cytokines in the ileum of piglets. **(A)** mRNA expression levels of *IFN-*α *and IFN-*β detected by qPCR in the ileum of piglets. **(B)** Protein expression levels of IL-1β, IL-6, and IL-8 detected by ELISA in the ileum of piglets. Values are mean and pooled SD, *n* = 8. ^a,b,c^Values with different letters for the same cytokine differ (*P* < 0.05).

As shown in Figure 4A, the mRNA expression levels of *IFN-*α and *IFN-*β were increased in the ileum of PEDV-infected piglets compared with the Control group. However, PR administration reduced (*P* < 0.05) the expression of *IFN-*α and *IFN-*β in PEDV-infected piglets. Similar patterns were also observed for the protein levels of IL-1β, IL-6, and IL-8 (Figure 4B).

### Identification and Comparison of DRPs

**Figure 5:**
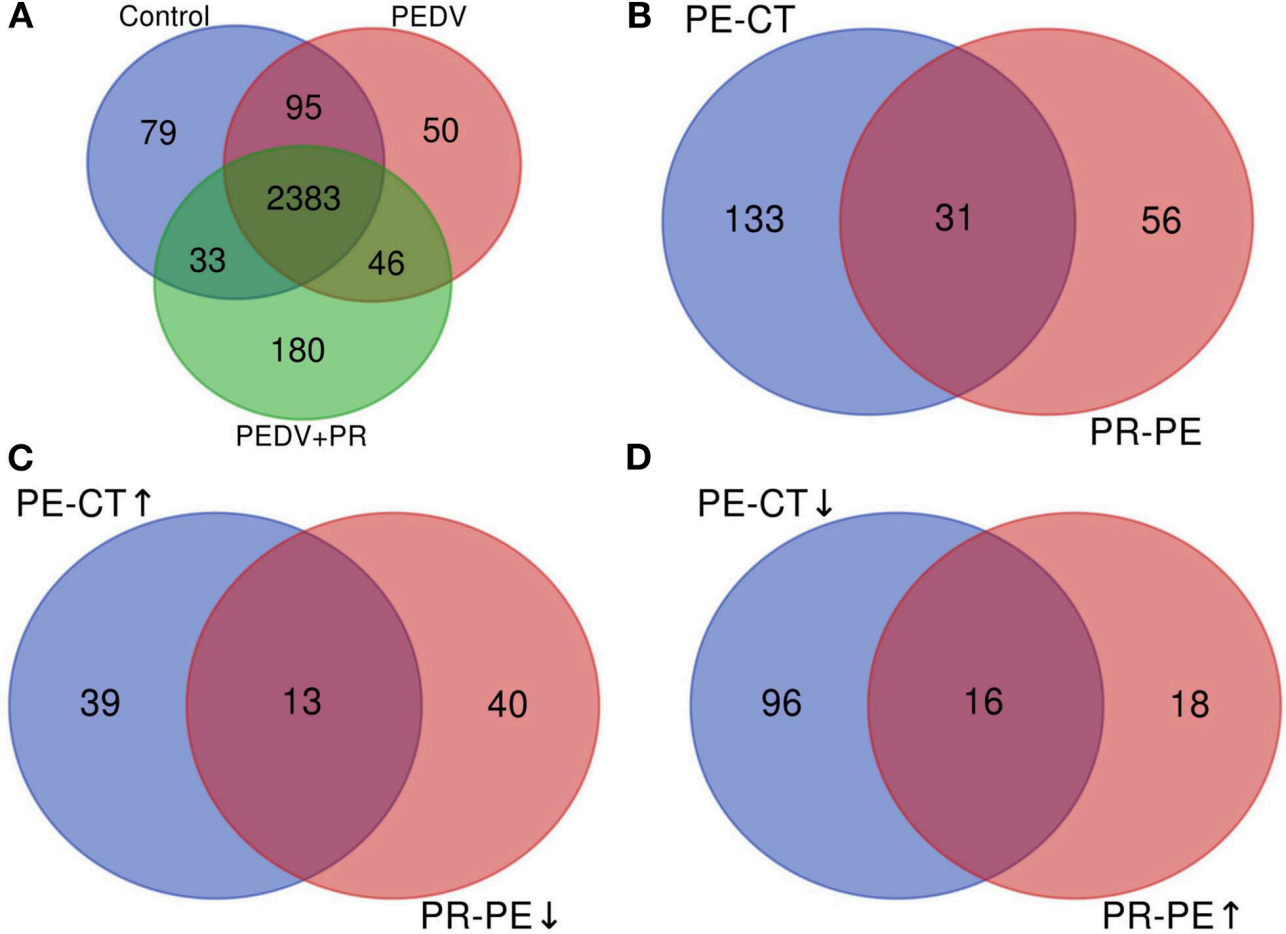
Overview of the upregulated and downregulated proteins identified in the small intestine of PEDV-infected piglets. **(A)** The Venn diagram shows the total numbers of identified and quantified proteins. **(B)** The total numbers of differentially regulated proteins (DRPs) in the PE-CT and PR-PE groups. **(C)** The overlapped numbers of both upregulated proteins in the PE-CT groups and downregulated proteins in the PR-PE groups. **(D)** The overlapped numbers of both downregulated proteins in the PE-CT groups and upregulated proteins in the PR-PE groups. PE-CT, the PEDV group vs. the Control group; PR-PE, the PEDV + PR group vs. the PEDV group. *n* = 4; ↑, upregulated; ↓, downregulated.

A total of 2,590, 2,574, and 2,642 proteins were identified and quantified in the control, PEDV, and PEDV + PR groups, respectively. Compared to the non-infected Control group, 52 proteins were upregulated and 112 proteins were downregulated in the PEDV group (FC > 1.5 or <0.67, *P* < 0.05). A total of 34 proteins were upregulated and 53 proteins were downregulated (FC > 1.5 or <0.67, *P* < 0.05) in the PEDV + PR group in comparison with the PEDV group. In addition, among these DRPs, 29 proteins were restored after PR intervention, including 13 downregulated and 16 upregulated proteins (Figure 5; Table S1).

### Bioinformatics Analysis of DRPs

**Figure 6:**
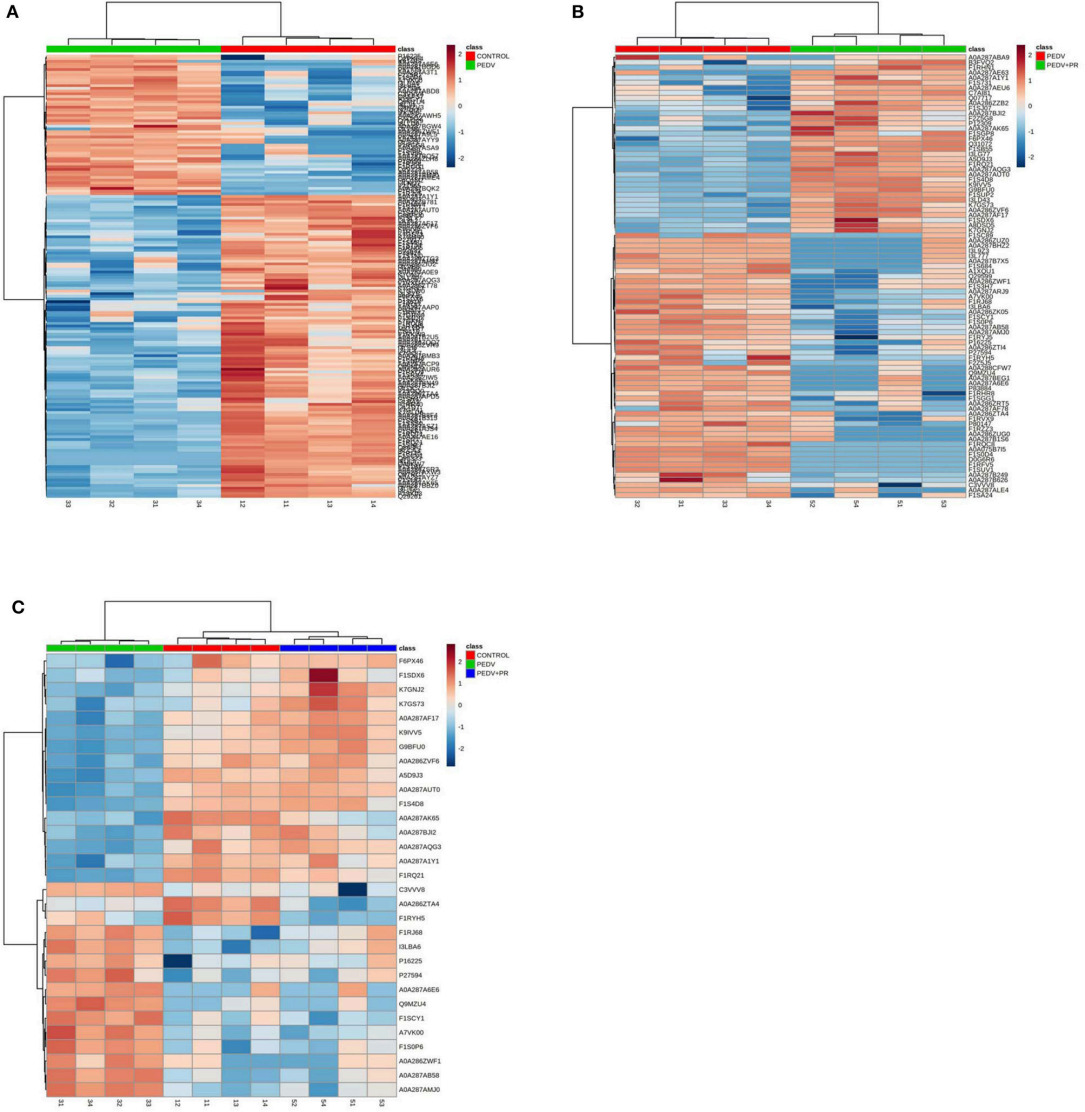
Heat-maps of differentially regulated proteins (DRPs). **(A)** The PEDV group vs. the Control group. **(B)** The PEDV + PR group vs. the PEDV group. **(C)** DRPs among three groups. *n* = 4.

**Figure 7:**
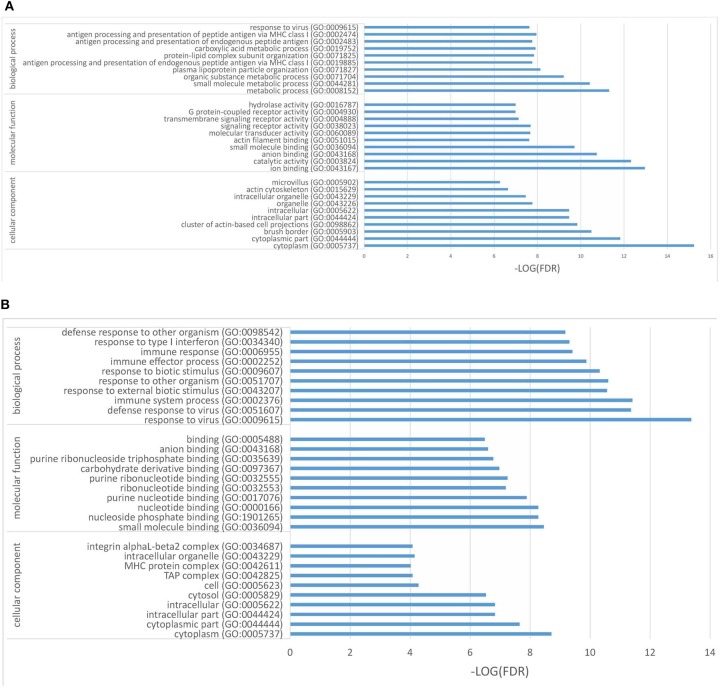
Differentially regulated proteins (DRPs) were categorized into cellular component (CCs), molecular function (MFs), and biological process (BPs) based on the GO analysis. **(A)** The PEDV group vs. the Control group. **(B)** The PEDV + PR group vs. the PEDV group. *n* = 4.

**Figure 8:**
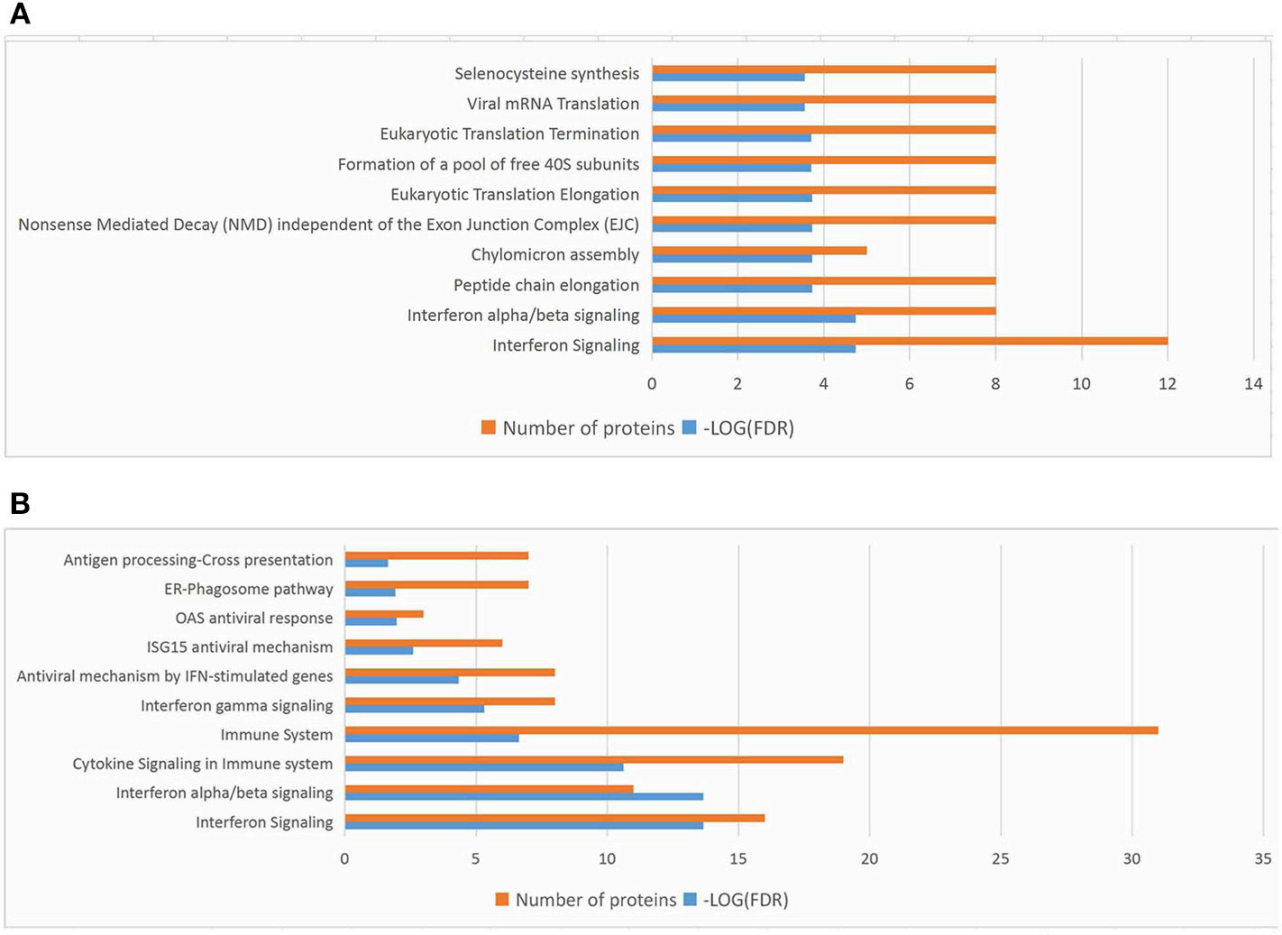
Top 10 significant pathways in PEDV or PEDV + PR piglets based on the reactome pathway analysis of the differentially regulated proteins (DRPs). **(A)** The PEDV group vs. the Control group. **(B)** The PEDV + PR group vs. the PEDV group. *n* = 4.

**Figure 9:**
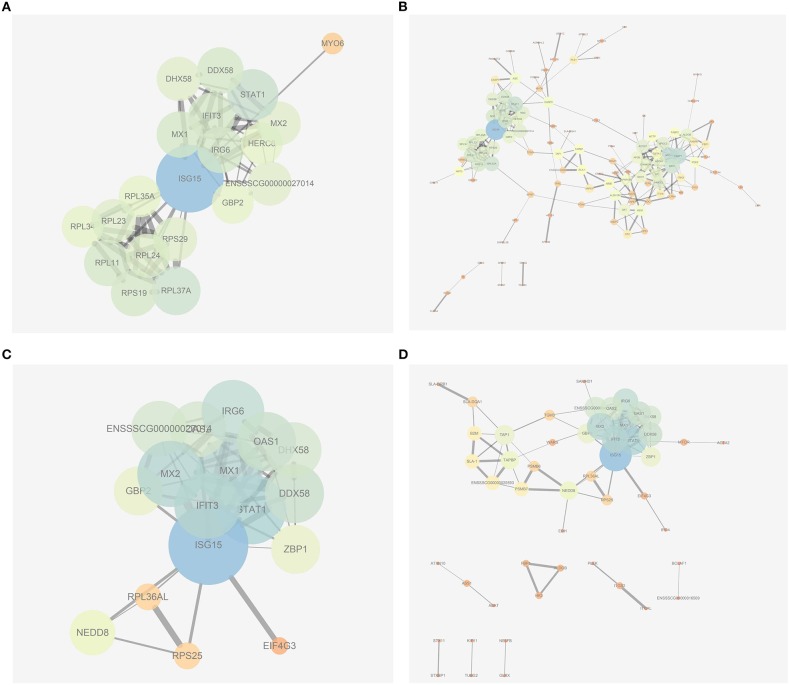
Protein–protein interaction networks built by using the online software String and Cytoscape. **(A)** The most significant part of networks from the PEDV group vs. the Control group. **(B)** An overview of networks from the PEDV group vs. the Control group. **(C)** The most significant part of networks from the PEDV + PR group vs. the PEDV group. **(D)** An overview of networks from the PEDV + PR group vs. the PEDV group. *n* = 4.

In the present work, heat-maps were generated on all filtered proteins, and differences in protein expression were observed when the PEDV group was compared to the Control group or the PEDV + PR group (Figures 6A,B). Similar patterns of protein expression were found for the Control group and the PEDV + PR group (Figure 6C). To further identify the functional characterization, DRPs were analyzed on the basis of GO categories: cellular component (CCs), molecular function (MFs), and biological process (BPs) (40, 41). As shown in Figure 7 and Table S2, BP analysis (FDR < 0.05) revealed that the DRPs associated with metabolic processes were enriched between the PEDV and Control groups. However, after PR intervention, the terms representing virus-related responses were most significant compared to the PEDV group. Meanwhile, the reactome pathway analysis identified that these DRPs were mainly involved in interferon signaling (Figure 8; Table S3). The protein–protein interaction networks also revealed that proteins related to interferon signaling interacted closely to fulfill their biological functions (Figure 9; Table S4).

### Validation of the Proteomic Analysis

**Figure 10:**
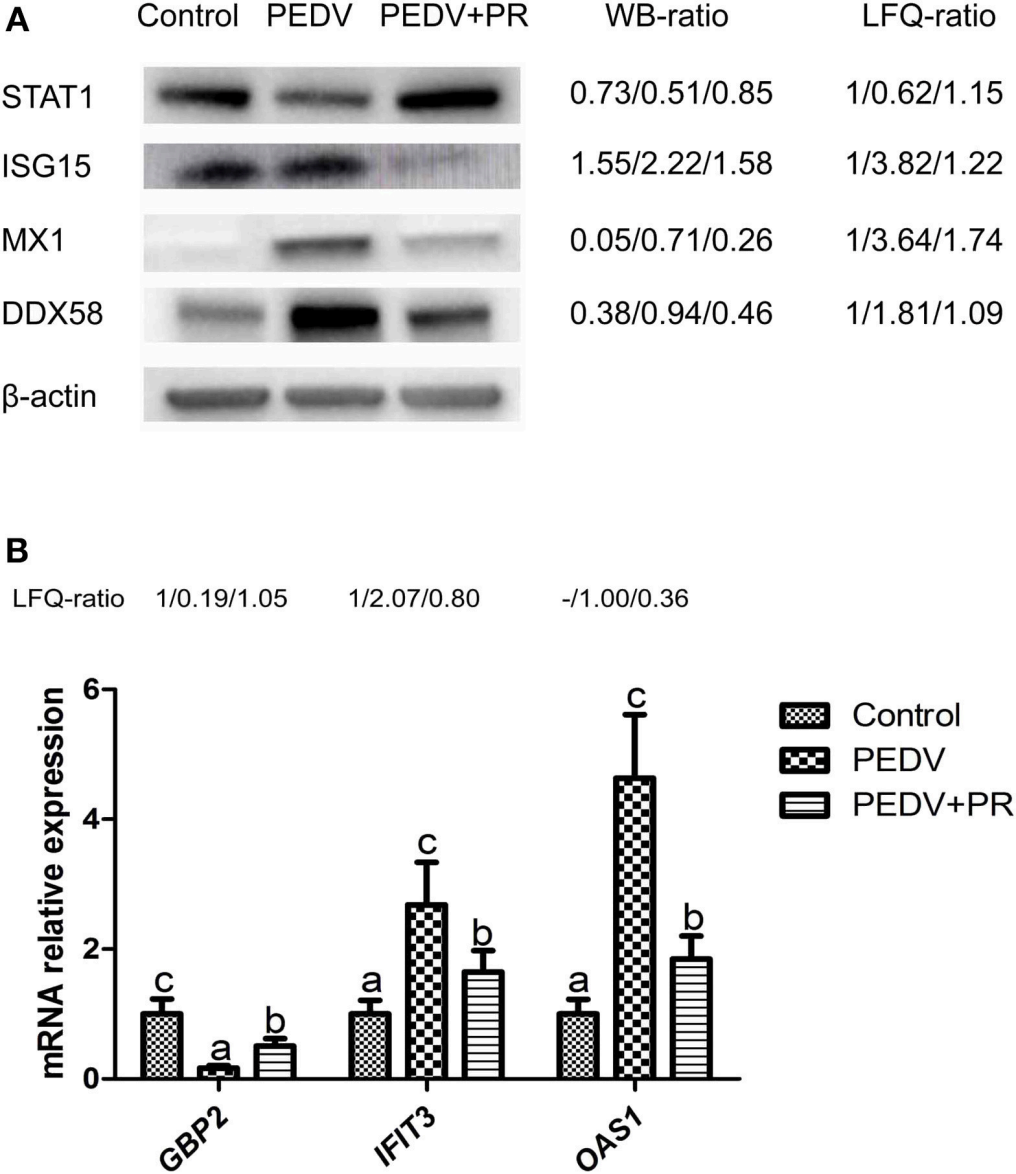
Validation of differentially regulated proteins (DRPs) by Western blot and qRT-PCR analyses. **(A)** Western blot. **(B)** qRT-PCR. The WB ratio (Western blot ratio) was calculated based on the relative intensity. Beta-actin was used as the loading control. The LFQ ratios for PEDV/Control and PEDV + PR/Control obtained by MS analysis are shown on the right. Values are mean and SD, *n* = 8. ^a,b,c^Values with different letters for the same protein are different (*P* < 0.05). *n* = 8. -: This protein in the Control group was undetected in LFQ.

To validate the results of the proteomic analyses, four proteins (STAT1, ISG15, MX1, DDX58) were analyzed using the Western blot technique. In accordance with the proteomic data, the expression of ISG15, MX1, and DDX58 was increased, but the expression of STAT1 was decreased post PEDV infection. PR intervention decreased (*P* < 0.05) the expression of ISG15, MX1, and DDX58, while it increased the expression of STAT1, compared to that of the PEDV group (Figure 10A). Owing to the lack of the appropriate antibodies for porcine tissues, qRT-PCR was alternatively used to validate the expression of *IFIT3, OAS1*, and *GBP2*. Likewise, a consistent trend of gene expression was observed among these groups of piglets (Figure 10B). The fold changes in the protein abundances among the three different groups of piglets, assessed by the LC-MS/MS analysis, are shown in Figure 10. These results indicated that the proteomics data are highly reliable.

### Regulation of Intestinal NF-κB Activation by PR in PEDV-Infected Piglets

**Figure 11:**
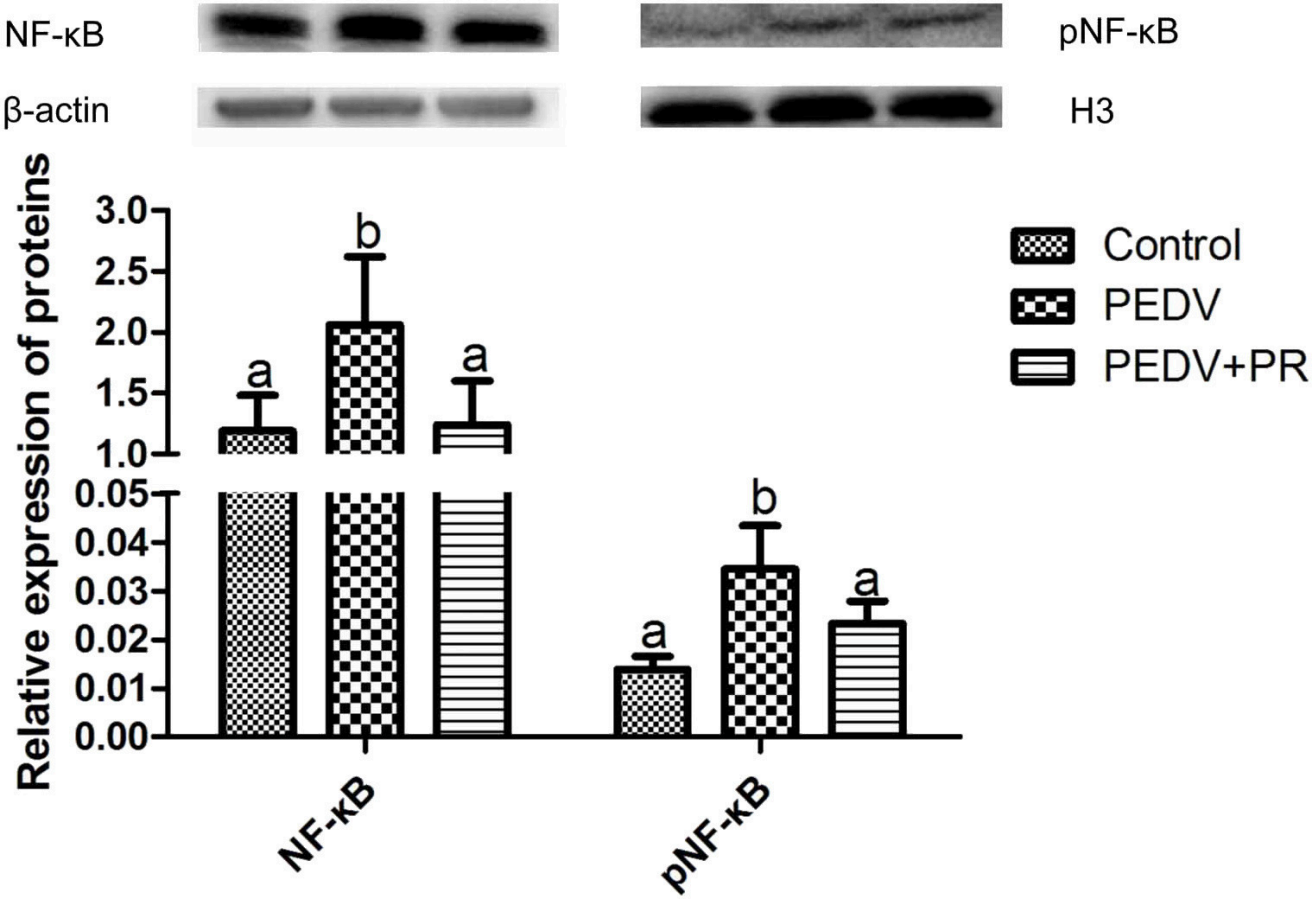
Relative expression of NF-κB and pNF-κB in the ileum by the Western blot analysis. Values are mean and SD, *n* = 8. ^a,b^Values with different letters for the same protein are different (*P* < 0.05). *n* = 8.

NF-κB is a key transcriptional factor in cytokine production, which may facilitate PEDV pathogenesis (42, 43). As shown in Figure 11, PEDV infection increased (*P* < 0.05) the expression of NF-κB and pNF-κB, while both NF-κB, and pNF-κB expressions were almost fully restored to the control levels by PR administration, indicating that PR could inhibit PEDV-induced NF-κB activation *in vivo*.

## Discussion

PEDV is the major etiological agent for the recent outbreaks of piglet diarrhea that has caused devastating economic losses to the swine industry worldwide. Traditional vaccine strategy cannot either provide a full protection for piglets or ensure a satisfying control of PEDV infection and spreading. Therefore, the development of safe, effective, and inexpensive antiviral drugs remains a great challenge to modern medicine. PR is a natural isoflavonoid extracted from Gegen in the traditional Chinese herb medicine. As a potential antiviral drug, effects of PR on PEDV infection are unknown. To date, although the proteomic technique has been widely applied to virus–host interaction studies, little information is available about the protein profiles of piglets infected with PEDV. In the current work, we determine the effect of PR on PEDV both *in vitro* and *in vivo* with the goal of elucidating the underlying mechanisms by a proteomic analysis.

PEDV can cause acute and watery diarrhea, vomiting, dehydration, and high mortality in neonatal piglets (43), and its adverse effect on growth performance of piglets was demonstrated by Curry et al. (44). Consistently, in the present study, PEDV infection resulted in a significant reduction in piglet ADG, but PR administration substantially alleviated the reduction of ADG, indicating that PR has a positive effect on the growth performance of PEDV-infected piglets. Although the reduction in diarrhea rate was not statistically significant due to the small sample size, the decreasing trend in diarrhea rate and the weaker clinical symptoms indicated that the piglets in the PEDV + PR group were recovering better from illness. Similarly, dietary supplementation with soy isoflavone increased ADG and reduced the incidence of diarrhea in weaned piglets challenged with lipopolysaccharide (45). These results support the notion that isoflavonoid could be used to treat diarrhea (46). Our findings further demonstrated that PR could alleviate reduction in the growth performance of PEDV-infected piglets.

In the present study, the mRNA levels of genes that encoded for the *N* and *M* proteins of PEDV were decreased regardless of whether PR was administered after the entry of the virus into Vero cells *in vitro* or before PEDV infection in piglets. Similar results were found for the N and M protein levels, which were determined by the proteomic analysis (data not shown). These results indicated that PR could inhibit the replication of PEDV at different stages of its life cycle. Nevertheless, additional comprehensive studies are needed to verify this notion. In the current work, *in vitro* studies were performed with monkey cells and *in vivo* studies with piglets. Although they are different species, both of them are highly susceptible to PEDV, and PR could inhibit the replication of PEDV in both species. This suggests that PR was effective against PEDV *in vitro* and *in vivo*. Jejunal and ileal villus enterocytes are the main targets of PEDV replication (47), and PEDV also appears in feces and mesenteric lymph nodes (48). Consistently, we found that the piglet small intestine was the main target of PEDV. Intriguingly, the mRNA levels of *N* and *M* genes were extremely high in the ileum of piglets, even higher than those in the jejunum. Meanwhile, the mRNA levels of *N* and *M* genes were also high in colonic contents. Importantly, PR administration decreased the expression of the viral genes in the ileum, jejunal, colon, and mesenteric lymph nodes, indicating that PR plays an antiviral role in a large part of the gut. Previous studies showed that PR inhibited HIV-1 replication and human respiratory syncytial virus activities *in vitro* (20, 21). Some medicinal herbal extracts, such as oleanane triterpenes from the flowers of *Camellia japonica, Sophorae radix, Acanthopanacis cortex, Sanguisorbae radix*, and *Torilis fructus*, can inhibit PEDV replication (49, 50). To date, there is little information regarding an anti-PEDV effect of PR. To the best of our knowledge, this is the first study demonstrating that PR inhibited PEDV replication both *in vitro* and *in vivo*.

Inflammation and disease progression are initiated by pro-inflammatory cytokines and chemokines during viral infection (51). A previous study showed that the mRNA levels for pro-inflammatory cytokines (e.g., *IL-1*β, *IL-6, IL-8*, and *TNF-*α) were increased in Vero cells after PEDV infection (52). *MCP1/CCL2*, which is a chemokine, was also increased in response to PEDV infection (53). Likewise, we found that *IL-8, TNF-*α, and *MCP1* expressions were increased post PEDV infection in Vero cells and that the concentrations of IL-1β, IL-6, and IL-8 were increased in the ileum of PEDV-infected piglets, further substantiating the notion that PEDV could induce inflammatory response. Interestingly, inflammation appeared to be alleviated in the PEDV + PR group, as indicated by the decreased expression of the inflammatory cytokines. This result indicated that PR possessed an anti-inflammatory effect, which is consistent with the previous reports that the expression of genes for inflammatory mediators (e.g., *IL-1*β, *IL-6*, and *TNF-*α) were reduced by PR administration under inflammatory conditions (11, 54). Collectively, these findings support the view that PR can mitigate inflammation induced by PEDV infection *in vitro* and *in vivo*.

Cluster analyses are usually used to build groups of functionally related genes or proteins, thereby interpreting and identifying DRPs among different groups. In this study, nearly all of the overlapped DRPs (29/31) between the PEDV and Control groups or between the PEDV + PR and PEDV groups were reversely regulated by PR intervention, which could be clearly visualized by the heat-maps. Several functional enrichment analyses highlighted the importance of the interferon signaling pathway. Specifically, IFN-α/β and several ISGs were selectively upregulated at mRNA or protein levels in response to PEDV infection. This result is inconsistent with the previous report that PEDV antagonized interferon response (10, 55). Because almost all of the previous studies were conducted *in vitro*, the inconsistency may be attributed to the fact that results of *in vitro* experiments may not accurately reflect the situation *in vivo*. Moreover, an *in vivo* study performed by Annamalai et al. (56) demonstrated that serum IFN-α could be augmented at the early stage of PEDV infection. Of particular interest, PR administration appeared to regulate the interferon signaling pathway by maintaining the expression of IFN-α/β and ISGs at their normal physiological levels. It is noteworthy that GBP2, interferon-inducible guanylate-binding protein 2, was markedly upregulated in the PEDV + PR group. GBPs play an important role in antiviral infection (57). Further studies are warranted to determine whether PR exerts an antiviral effect by regulating GBP2 expression. Overall, these results indicated that PR regulated the interferon signaling pathway in PEDV-infected piglets.

The NF-κB pathway plays a central role in the regulation of immune responses. Such a function of NF-κB in PEDV infection is supported by several lines of evidence (32, 43, 58). Our results showed that PEDV induced NF-κB activation *in vivo* and that PR supplementation inhibited the NF-κB activation, which may contribute to the decrease in the expression of interferon-related genes and the production of cytokines. This is consistent with the previous findings that PR has an anti-inflammatory effect *in vitro* and *in vivo* (59, 60). Therefore, we suggest that PR is beneficial for restoring immune homeostasis after PEDV infection by inhibiting NF-κB activation.

## Conclusion

PR inhibited PEDV replication both *in vitro* and *in vivo* and alleviated the decrease of growth performance in piglets. Additionally, the quantitative proteomic analysis revealed that PR administration regulated the interferon signaling pathway in PEDV-infected piglets. Furthermore, PR attenuated NF-κB activation in the ileum. Taken together, these findings suggest that PR protected against PEDV infection via regulating the interferon and NF-κB signaling pathway. Our results are expected to aid in the development of effective antiviral feed additives and also have important implications for the production of veterinary drugs and human medicine.

## Data Availability Statement

All datasets generated for this study are included in the article/Supplementary Material.

## Ethics Statement

The animal study was reviewed and approved by Animal Care and Use Committee at Wuhan Polytechnic University.

## Author Contributions

MW, QZ, DY, YH, and GW were responsible for the study design, proteome, database search, data interpretation, drafting the paper, final approval, and agreement to be accountable. TW, HC, and SG were responsible for the study design, proteome, data interpretation, critical revision, final approval, and agreement to be accountable. SL, CJ, LW, and DZ were responsible of the animal experiments, data interpretation, critical revision, final approval, and agreement to be accountable.

### Conflict of Interest

The authors declare that the research was conducted in the absence of any commercial or financial relationships that could be construed as a potential conflict of interest.

## Abbreviations

GBPs: guanylate-binding proteins
HPRT1: hypoxanthine phosphoribosyltransferase 1
IFIT3: interferon-induced protein 3
IL-1β: interleukin−1β
IL-8: interleukin-8
ISGs: interferon-stimulated genes
MCP-1: monocyte chemotactic protein 1
NF-κB: nuclear factor kappa-B
OAS1: 2′-5′oligoadenylate synthetase 1
PEDV: porcine epidemic diarrhea virus
PEDV-M: membrane protein (porcine epidemic diarrhea virus)
PEDV-N: nucleocapsid protein (Porcine epidemic diarrhea virus)
RPL4: ribosomal protein L4
TCID_50_: 50% tissue culture infectious dose
TNF-α: tumor necrosis factor α.
